# Temporal and spatial dynamics of trypanosomes infecting the brush-tailed bettong (*Bettongia penicillata*): a cautionary note of disease-induced population decline

**DOI:** 10.1186/1756-3305-7-169

**Published:** 2014-04-07

**Authors:** Craig K Thompson, Adrian F Wayne, Stephanie S Godfrey, RC Andrew Thompson

**Affiliations:** 1 School of Veterinary and Life Sciences, 90 Murdoch University, South Street, Western Australia 6150, Australia; 2 Science Division, Department of Parks and Wildlife, Manjimup WA 6258, Australia

**Keywords:** *Trypanosoma vegrandis*, *T. copemani*, Woylie, *Bettongia penicillata*, Wildlife disease, Interspecific competition

## Abstract

**Background:**

The brush-tailed bettong or woylie (*Bettongia penicillata*) is on the brink of extinction. Its numbers have declined by 90% since 1999, with their current distribution occupying less than 1% of their former Australian range. Woylies are known to be infected with three different trypanosomes (*Trypanosoma vegrandis*, *Trypanosoma copemani* and *Trypanosoma* sp. H25) and two different strains of *T. copemani* that vary in virulence. However, the role that these haemoparasites have played during the recent decline of their host is unclear and is part of ongoing investigation.

**Methods:**

Woylies were sampled from five locations in southern Western Australia, including two neighbouring indigenous populations, two enclosed (fenced) populations and a captive colony. PCR was used to individually identify the three different trypanosomes from blood and tissues of the host, and to investigate the temporal and spatial dynamics of trypanosome infections.

**Results:**

The spatial pattern of trypanosome infection varied among the five study sites, with a greater proportion of woylies from the Perup indigenous population being infected with *T. copemani* than from the neighbouring Kingston indigenous population. For an established infection, *T. copemani* detection was temporally inconsistent. The more virulent strain of *T. copemani* appeared to regress at a faster rate than the less virulent strain, with the infection possibly transitioning from the acute to chronic phase. Interspecific competition may also exist between *T. copemani* and *T. vegrandis*, where an existing *T. vegrandis* infection may moderate the sequential establishment of the more virulent *T. copemani.*

**Conclusion:**

In this study, we provide a possible temporal connection implicating *T. copemani* as the disease agent linked with the recent decline of the Kingston indigenous woylie population within the Upper Warren region of Western Australia. The chronic association of trypanosomes with the internal organs of its host may be potentially pathogenic and adversely affect their long term fitness and coordination, making the woylie more susceptible to predation.

## Background

Trypanosomes of humans and livestock have been intensely studied because of their potential to cause death, sickness and reduce economic gain. In contrast, the trypanosomes of Australian wildlife have been largely neglected. Of the approximately 120 described *Trypanosoma* species known to infect mammals around the world by 1972, only 4% (4 native and 1 exotic) had been identified in native Australian mammals [1,2]. However, as an increasing number of Australian mammal species suffer from the risk of extinction, there has been a renewed interest in the identification and understanding of these parasites [3-8]. The potential for diseases (such as those caused by trypanosomes) to reduce the fitness of wildlife hosts and influence their population decline is of particular concern [9,10], especially given recent evidence linking *Trypanosoma lewisi* with the extinction of two native rodent species (*Rattus macleari* and *Rattus nativitatis*) on Christmas Island [11-13].

**Figure 1:**
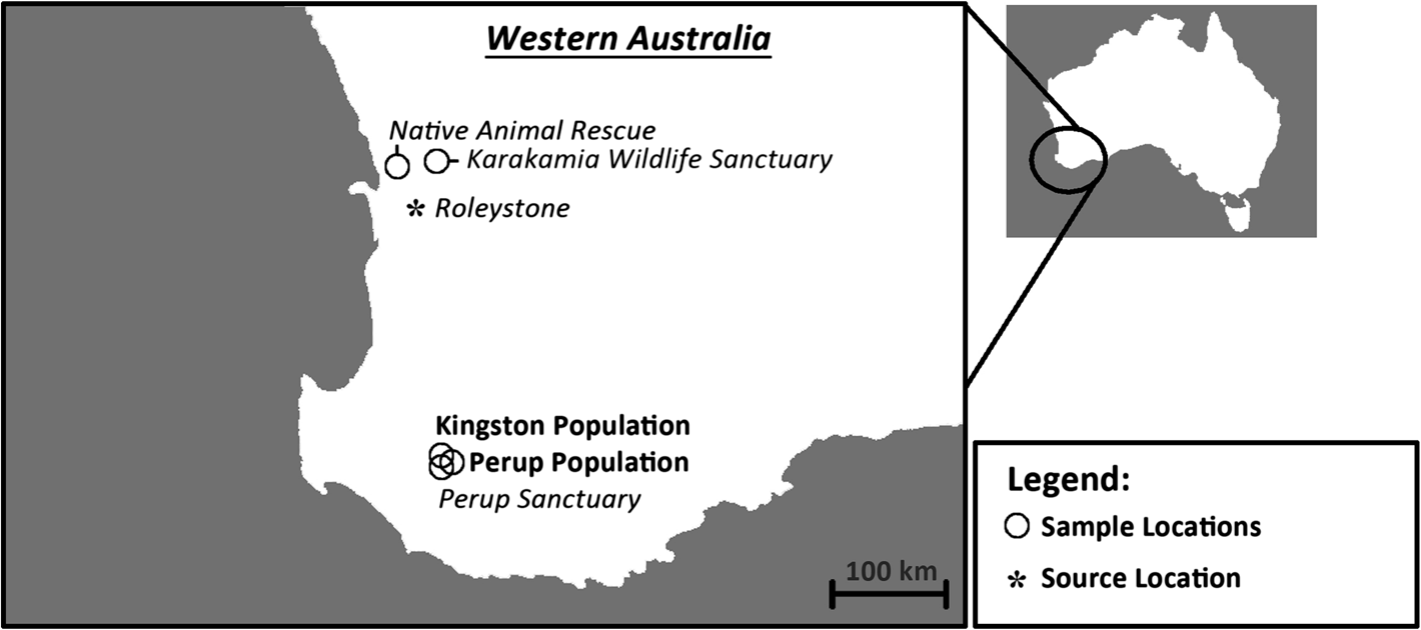
**Sample locations in Western Australia. ‘bold’** denotes an indigenous population and *‘italics’* denotes an enclosed population.

One such mammal on the brink of extinction is the critically endangered brush-tailed bettong or woylie (*Bettongia penicillata*), which is host to three different trypanosomes (*Trypanosoma vegrandis, Trypanosoma copemani* and *Trypanosoma* sp H25), two strains of *T. copemani* that vary in virulence (*T. copemani* P1 and P2) and an ectoparasitic tick (*Ixodes australiensis*) that may be a vector of *T. copemani* (with transmission possibly occurring via the faecal-oral route) [5,6,14]. Remaining indigenous woylie populations currently occupy less than 1% of their former range and are restricted to two locations in southern Western Australia (WA), namely Dryandra Woodland and the Upper Warren region (UWR) [15-21]. The UWR woylie population, which represent about 74% of the remaining indigenous individuals, have recently been identified as two genetically distinct and isolated populations; these include the Kingston population (KP) in the west and the Perup population (PP) to the east [22,23] (Figure 1). Up until recently, Tutanning Nature Reserve was considered the fourth small population of indigenous woylies; but it is now considered extinct [22].

In spite of conservation efforts, woylies have declined by approximately 90% at a species level since 1999 [22]. The reason(s) for the recent declines remain unknown; however, the spatio-temporal pattern to the declines is one of the lines of evidence that indicates that disease may have contributed by making woylies more vulnerable to predation or to other, as yet unidentified, stressors [9,10]. In an effort to ensure the survival of the species, large enclosures were constructed to allow ‘insurance populations’ of woylies to breed within a predator free environment. Two such examples of these enclosures include the newly constructed and stocked Perup Sanctuary (PS) (420 ha enclosure) and the well-established Karakamia Wildlife Sanctuary (KWS) (285 ha enclosure), both located in WA [6,10] (Figure 1).

The trypanosomes of the woylie are suspected of playing a role in the recent decline of their host. An association was identified linking the intracellular stage of *T. copemani* to the changes to the smooth and cardiac muscles of the woylie, with characteristics of infection reportedly similar to Chagas disease in humans [5]. In this study, we examined the spatial and temporal dynamics of *T. vegrandis, T. copemani* and *T.* sp. H25 infections in five different woylie populations in WA (Figure 1). The first study location was Native Animal Rescue (NAR), where a small captive colony was intensely monitored for two years. The remaining four locations (which include the neighbouring indigenous KP and PP within the UWR, the newly stocked PS, and KWS) were each monitored over a 19 month period. To improve our understanding of these trypanosomes, we monitored changes in infection dynamics over time, the interaction of co-infections and their potential to influence the recent population declines of the host.

## Methods

### Sample collection and preparation

Sheffield traps were used to capture woylies from five locations in WA (Figure 1). Traps were baited with a mixture of rolled oats, peanut butter and sardines and were set prior to dusk and checked at first light the following morning. The first study location was at NAR, where 16 woylies were sampled monthly between March 2010 and February 2013 (with no samples collected during January 2013). These 16 woylies were sourced from three locations; four from KP, four from PP (as below) and eight from a private enclosure at Roleystone (Figure 1). All eight of the Roleystone woylies were PCR negative to trypanosomes at the time of transfer (using PCR methods described below). The next two study locations were the KP and PP within the UWR. Both indigenous populations were sampled six separate times each between October 2010 and April 2012. Woylies caught during the second session at both of these locations were used to stock the newly constructed PS. Sequential sampling of woylies within the PS (the fourth study location) was conducted four additional times between February 2011 and April 2012. The final study location was at KWS, with woylies sampled five separate times between September 2010 and February 2012. Estimated and known stocking sizes of each population are presented in Table 1.

**Table 1:** Estimated and known stocking sizes of the five populations sampled

**Study populations**	**Estimated/Stocking size**
Kingston Population (KP)	Collectively (KP + PP), an estimated 8300 woylies in these two neighbouring populations*
Perup Population (PP)	
Perup Sanctuary (PS)	Stocked with 41 woylies
Karakamia Wildlife Sanctuary (KWS)	Estimated 500 woylies*
Native Animal Rescue (NAR)	Stocked with 16 woylies

*Source Wayne *et al.*[22].

The woylies were managed and handled using procedures formally approved by the Murdoch University Animal Ethics Committee (AEC number: W2350-10) and Department of Park and Wildlife Animal Ethics Committee (AEC number: DPaW 2010/36) in compliance with the Australian Code of Practice for the use of Animals for Scientific Purposes.

Individual woylies were identified by either an ear tag or a Permanent Integrated Transponder (PIT) number to ensure that blood was only extracted once per trapping session. Using a 25G × 5/8” needle and 1 ml syringe, 300 μl of blood was collected from the lateral caudal vein of each woylie and placed into a MiniCollect 1 ml EDTA tube (Greiner bio-one, Germany) to prevent clotting and kept at 4°C for DNA extraction and PCR. After blood collection, all woylies were released at the point of capture, except for 54 woylies captured in the UWR during November and December 2010.

Of these 54 woylies from the UWR, 41 woylies (9 KP and 32 PP woylies) were translocated to PS and were the founding individuals within this enclosure. Five (2 KP and 3 PP woylies) were translocated to the Perth Zoo as part of an unrelated project. The remaining eight individuals (4 PP and 4 KP woylies) were translocated to NAR after they were identified as being naturally infected with trypanosomes by microscopy [6].

In addition to the founding woylies at PS and NAR, ‘first-generation’ sub-adult woylies born within the enclosures were also sampled. Twenty-nine first-generation woylies were tested at PS, with an additional 29 tested at NAR. All of the first-generation woylies at NAR were relocated once they were independent of their mother, and were no longer part of this study.

On a single occasion at NAR, an injured adult woylie (origin: PP) was captured. This woylie had lost significant body mass and was euthanized following examination by qualified veterinary staff. Tissue samples were collected during autopsy and were taken from near the center of each organ before being stored in 70% ethanol.

### DNA extraction

Blood collected in EDTA tubes was used for genomic DNA extraction, along with tissue samples from the single dead woylie from NAR. DNA was extracted from 300 μl of host blood and 10–20 mg of host tissue using the Wizard® Genomic DNA Purification Kit (Cat# A1125) as per the protocol for whole blood extraction and animal tissue (Promega, Wisconsin USA). DNA was eluted in 60 μl of DNA rehydration solution and stored at −20°C prior to use. A negative control was included in each batch of DNA extractions that contained neither blood nor tissue.

### Species/clade-specific PCR

Three separate species/clade-specific nested PCR protocols were used to amplify the trypanosome 18S rDNA region. *Trypanosoma vegrandis* (TVEF, TVER, TVIF and TVIR) and *T. copemani* (S825F, SLIR, WoF and WoR) species-specific PCR primers and PCR reactions were used as previously described by Thompson *et al.*[6]. *Trypanosoma* sp. H25 clade-specific PCR primers (H25EF, H25ER, H25IF and H25IR) and PCR reactions were used as previously described by Botero *et al.*[5]. Four controls were used in every nested PCR and included the negative control from the DNA extraction, a primary and a secondary PCR negative control and PCR positive control. Each control was monitored to ensure reliability of results. PCR products were run on a 1.5% agarose gel using SYBR Safe Gel Stain (Invitrogen, California USA) and visualized by illumination with UV light.

### Analysis

For the analyses, data were sorted into seasons, with summer being December, January and February; autumn being March, April and May; winter being June, July and August; and spring being September, October and November.

A generalised linear mixed effects (glmer) model was used to test the effects of location, season and time on the infection state of woylies, separately for each of the different species of *Trypanosoma*. For each species of *Trypanosoma*, infection state (0 (uninfected) and 1 (infected)) was the dependent variable, and location and season were the independent factors. Individual ID was also included as a random effect to account for repeated measures on individuals, and the models had a binomial error distribution.

To investigate what factors influenced the incidence of new infections (changes in infection state) a generalised linear model (glm) was used to test the effects of location, season and prior infection status on the incidence of new infections. The last record for each individual was selected (so there were no repeated measures of individuals – the data were too over-dispersed to use the glmer models with a binomial error distribution), and categorised infection change as 1 for a new infection, and a 0 as no change or infection lost (as the factors influencing loss of infection in this analysis were not of interest). Infection change was the dependent variable, and location, and prior infection state with each of the other *Trypanosoma* species were included as independent factors in our analysis. A quasi-binomial error distribution was used and we tested the significance of the effects with a chi-squared test. For these analyses, only individuals that had been recaptured were included.

All the analyses were performed in the R statistical software [24], using the packages lme4 [25] and car [26].

## Results

### Overall trypanosome prevalences

During the 29 months of woylie sampling at the five locations in WA, a total of 881 blood samples were collected from 262 individuals (with these same blood samples contributing to the temporal and spatial results below). Of these 262 individuals, 134 (51.1%) were sampled only once, while the remaining 128 individuals (48.9%) were sampled two or more times.

**Figure 2:**
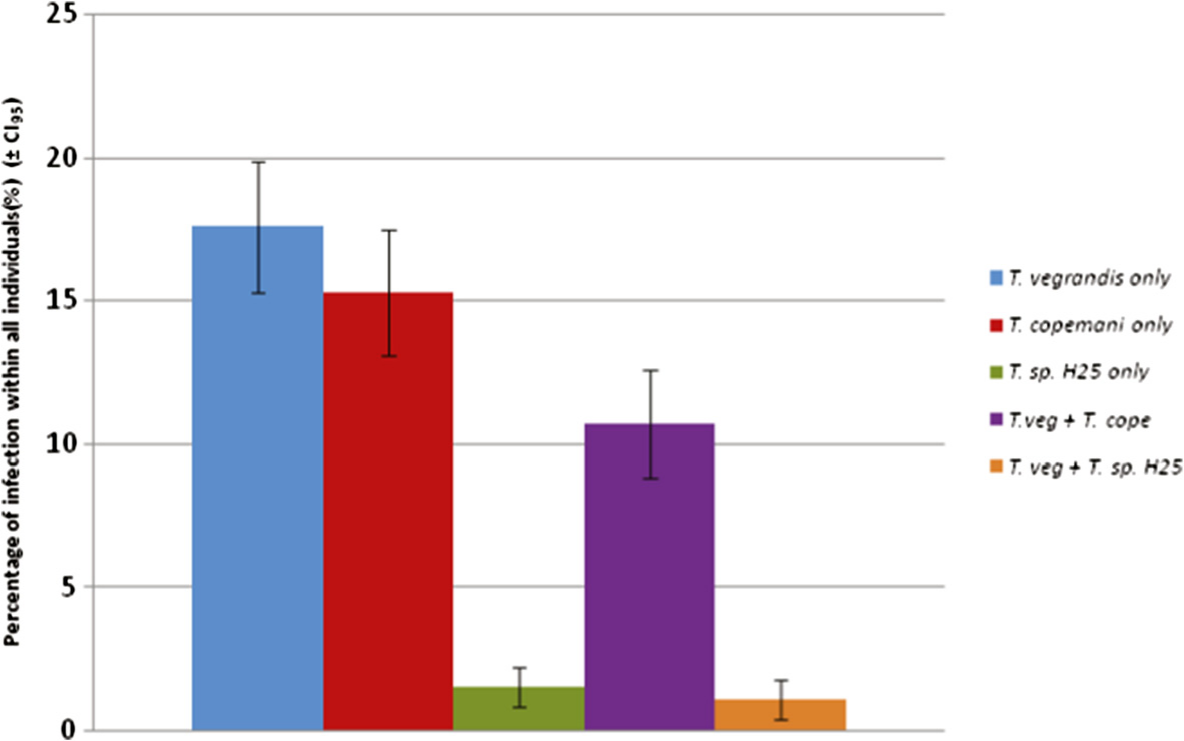
**Trypanosome prevalence of infected woylies only (N = 121) from the five study sites (with uninfected woylies not graphically represented).** Where a host was recorded as either infected or co-infected if it tested positive to a *Trypanosoma* sp. or multiple *Trypanosoma* spp. at least once during the study period.

Trypanosomes were identified, at least once, from 121 (46.2%, CI_95_ = 43.1 – 49.3%) individuals. For an individual, a single infection of *T. vegrandis* was more common than *T. copemani*, while *T.* sp. H25 was relatively uncommon (Figure 2). For an individual with a mixed infection, *T. vegrandis* and *T. copemani* co-infections were more common than *T. vegrandis* and *T.* sp. H25 (Figure 2). No individuals were identified with a co-infection of *T. copemani* and *T.* sp. H25, or with the three different trypanosomes.

### Temporal dynamics of trypanosome infections at NAR

During the intensive study of captive woylies at NAR, a total of 167 blood samples were collected from the eight trypanosome positive woylies that originated from KP and PP. Of these 167 samples, *T. vegrandis* was detected a total of 42 times (25.1%, CI_95_ = 21.8 – 28.5%) from five individuals only and *T. copemani* was detected a total of 131 times (78.4%, CI_95_ = 75.3 – 81.6%) from all eight individuals. *Trypanosoma* sp. H25 was absent from NAR.

The molecular identification of trypanosomes from the peripheral blood was not always temporally consistent at NAR. Following the initial identification of *T. copemani* from the eight woylies that were translocated from KP and PP, a total of 36 blood samples were collected (from five individuals only) that tested PCR negative to *T. copemani*. In 36% of these PCR negative cases, the sequential blood sample that followed tested positive to *T. copemani*, with these same five individuals each testing positive to *T. copemani* again after their initial negative result. Following the initial identification of *T. vegrandis* from five of the eight woylies that originated from KP and PP, a total of 45 blood samples were collected that tested PCR negative to *T. vegrandis.* In 38% of these PCR negative cases, the sequential blood sample that followed tested positive to *T. vegrandis*, with four of these five individuals testing positive to *T. vegrandis* again after their initial negative result.

**Figure 3:**
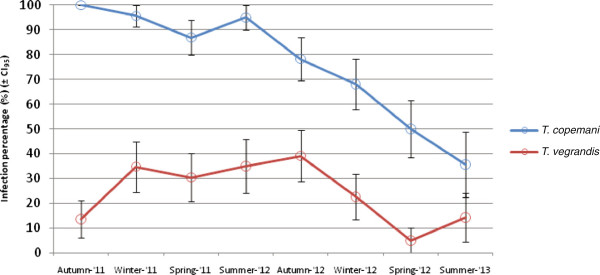
**Prevalence of *T. vegrandis *and *T. copemani *from the peripheral blood of the eight infected woylies at NAR during successive seasons.** Where each of the eight hosts was tested up to three times per season for both trypanosomes species, and the mean infection state (0(uninfected) and 1(infected)) was calculated for each season (± CI_95_).

**Figure 4:**
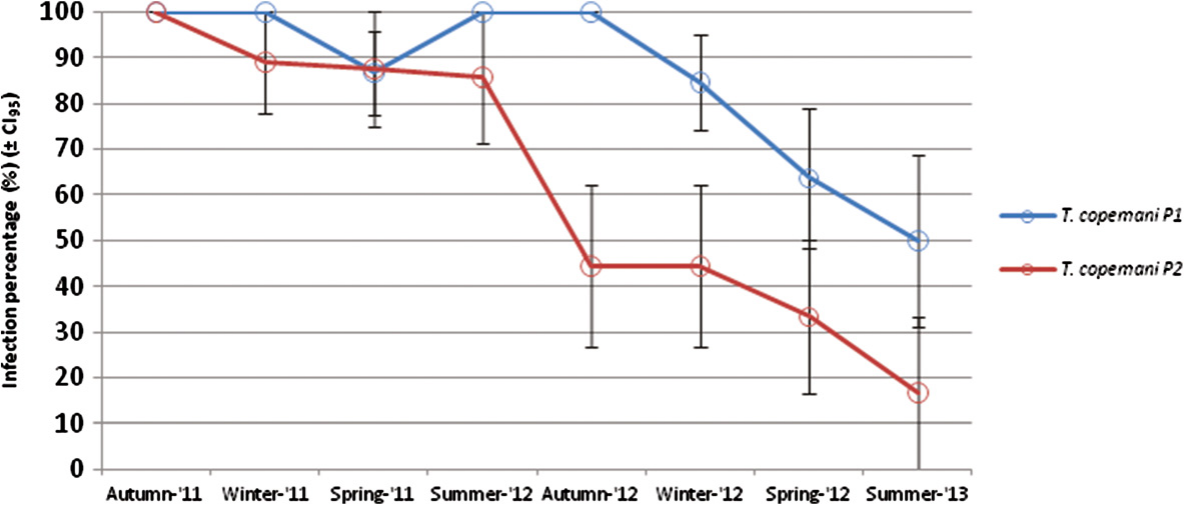
**Decline in prevalence of *T. copemani *P1 and P2 from the peripheral blood of the eight infected woylies at NAR during successive seasons.** Where each of the eight hosts was tested up to three times per season for *T. copemani*, and the mean infection state (0 (uninfected) and 1 (infected)) was calculated for each season (± CI_95_).

The prevalence of *T. copemani* positive blood samples was observed to decline with each passing season (*X*^*2*^ = 9.31, df = 1, *P =* 0.002), and was significantly different to *T. vegrandis* (*X*^*2*^ = 46.6, df = 1, *P <* 0.001) (Figure 3). When the eight infected woylies at NAR were separated into the two morphological phenotypes of *T. copemani* (where *T. copemani* P1 and P2 corresponds to Clade A Genotypes 1 and 2 respectively [5,6]) there was a significant effect of time on *T. copemani* P1 and P2 infection prevalence (*X*^*2*^ = 23.67, df = 1, *P <* 0.001), but there was no significant interaction between phenotypes and time (*X*^*2*^ = 0.80, df = 1, *P* = 0.371). Both phenotypes were observed to decline with each passing season, with the rate of decline appearing faster for *T. copemani* P2 than that of *T. copemani* P1 (Figure 4).

**Figure 5:**
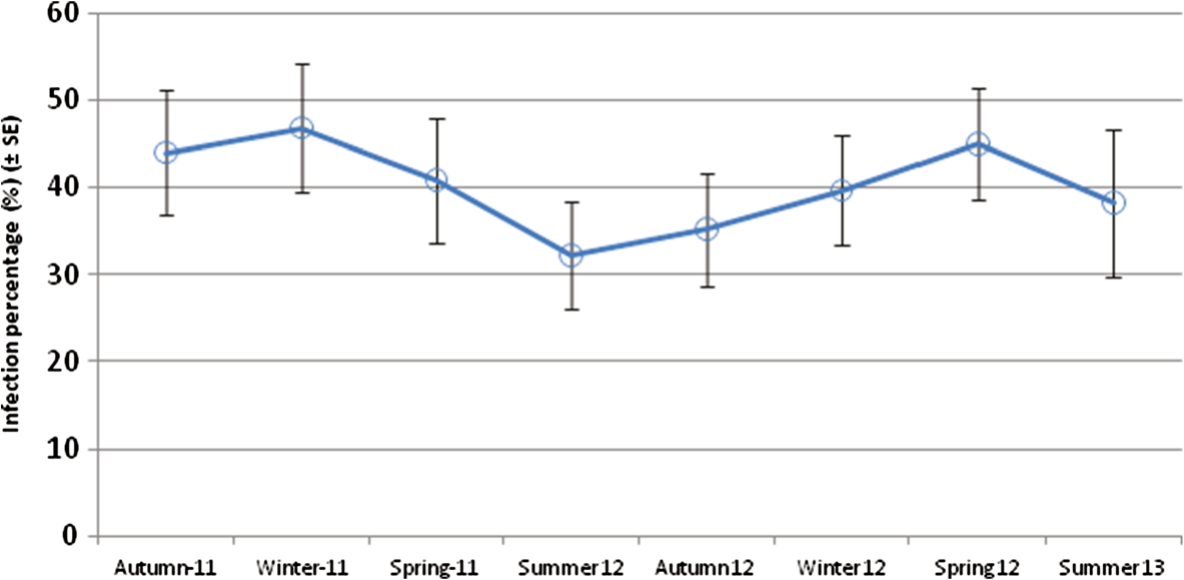
**Overall trypanosome prevalence (*T. vegrandis *and *T. copemani*) from the peripheral blood of 45 woylies at NAR during successive seasons.** Where each of the 45 hosts was tested up to three times per season for both trypanosomes species, and the mean infection state (0 (uninfected) and 1 (infected)) was calculated for each season (± CI_95_).

Of the remaining 37 woylies at NAR (8 Roleystone and 29 first-generation), the initial blood sample collected from each individual was PCR negative to the three different trypanosomes. Over the course of this investigation, five woylies that originated from Roleystone and seven first-generation woylies later tested positive to *T. copemani*. When taking into account the prevalence of trypanosome infections for all 45 woylies sampled at NAR, no significant linear relationships were evident on seasonality or time for either *T. vegrandis* (seasonality: *X*^*2*^ = 2.14, df = 3, *P =* 0.543; time: *X*^*2*^ = 0.60, df = 1, *P =* 0.439) or *T. copemani* (seasonality: *X*^*2*^ = 1.79, df = 3, *P =* 0.616; time: *X*^*2*^ = 0.44, df = 1, *P =* 0.505). The overall prevalence of trypanosome positive blood samples (*T. vegrandis* and *T. copemani*) was temporally inconsistent with the passing of each season (Figure 5).

Both *T. vegrandis* and *T. copemani* were detected from different organs of the single euthanised woylie from NAR. The organs tested for the three different trypanosomes are listed in Table 2.

**Table 2:** ***Trypanosoma vegrandis*, *T. copemani *** **and *T. *** **sp. H25 infected organs of the euthanised woylie at NAR**

**Organs**	***T. vegrandis***	***T. copemani***	***T. *** **sp. H25**
Blood	√	√	x
Bone marrow	√	√	x
Brain	√	√	x
Diaphragm	√	x	x
Heart	√	√	x
Kidney	√	√	x
Liver	√	√	x
Lung	√	√	x
Oesophagus	√	x	x
Skeletal muscle	√	x	x
Spinal cord	√	√	x
Spleen	√	√	x
Tongue	√	√	x

### Temporal dynamics of trypanosome infections at KP, PP, PS and KWS

Of the woylies caught two or more times from KP, PP, PS and KWS, eight individuals (four from PP, two from PS, and two from KWS) tested simultaneously positive to *T. vegrandis* and *T. copemani* after testing negative to trypanosomes in the previous sample period; two individuals (both from PP) tested positive to *T. vegrandis* and *T. copemani* after testing positive to *T. copemani* only the sample period prior; and five individuals (two from PP, and three from KWS) tested positive to both *T. vegrandis* and *T. copemani* upon their initial blood sample. Overall, there was a significant effect of the initial *T. vegrandis* infection on the subsequent likelihood of a co-infection with *T. copemani* (*X*^*2*^ = 3.16, df = 95, *P* = 0.009) when taking into account all of the *T. vegrandis* infected individuals caught two or more times during this study.

All nine woylies that were transferred from KP to PS tested negative to trypanosomes at the time of translocation. During the following 16 months, only one of these nine woylies tested positive for trypanosomes; this being a single identification of *T. copemani*. Of the 32 woylies transferred from PP to PS, 26 of them tested negative to trypanosomes at the time of translocation. When re-tested at a later date, thirteen of these woylies tested positive to trypanosomes; three were infected with *T. vegrandis* only, five with *T. copemani* only, three with *T.* sp. H25 only and two individuals tested simultaneously positive to *T. vegrandis* and *T. copemani*.

### Spatial variation of trypanosome infections

Prevalence of trypanosome infections from each of the five study sites are presented in Table 3. The prevalence of *T. vegrandis* was significantly different between sites PP, PS and KWS (*X*^*2*^ = 65.19, df = 2, *P <* 0.001), as was *T. copemani* between sites KP, PP, PS and KWS (*X*^*2*^ = 36.63, df = 3, *P <* 0.001). Of the five sites, the indigenous KP had the lowest prevalence of trypanosome infection, with only 5% of the population infected with *T. copemani*. In contrast, the neighbouring PP population had the highest prevalence of *T. copemani*, with 45% of the population infected with this trypanosome. KWS had the highest prevalence of *T. vegrandis*, with over 73% of the enclosed population infected with this trypanosome. Of those infected individuals at KWS, 100% of them were infected with *T. vegrandis*.

**Table 3:** **Prevalence (± CI**_**95**_**) of trypanosome infections at the five different study locations**

**Location**	**Individuals sampled**	**Uninfected individuals**	***T. vegrandis***	***T. copemani***	***T. *** **sp. H25**	***T. vegrandis & T. copemani***	***T. vegrandis & T. *** **sp. H25**
NAR	45	55.6% (48.1 – 63.0)	0%	33.3% (26.2 – 40.4)	0%	11.1% (6.4 – 15.85)	0%
KP*	43	93.0% (89.1 – 97.0)	0%	4.7% (1.4 – 7.9)	2.3% (0.0 – 4.7)	0%	0%
PP*	86	53.5% (48.1 – 58.9)	1.1% (0.0 – 2.3)	25.6% (20.8 – 30.3)	0%	19.8% (15.4 – 24.1)	0%
PS	70	71.4% (66.0 – 76.9)	2.9% (0.9 –4.9)	18.5% (13.9 – 23.3)	4.3% (1.8 –6.7)	2.9% (0.9 –4.9)	0%
KWS	67	22.4% (17.3 – 27.5)	64.2% (58.3 – 70.1)	0%	0%	8.9% (5.4 – 12.5)	4.5% (1.9 – 7.0)

*KP + PP = UWR.

## Discussion

Almost half of the individual woylies tested during this study were infected with trypanosomes, with a varying spatial pattern of infection identified from each of the study locations. More woylies at the UWR (KP and PP) were infected with *T. copemani* than at KWS, while all of the infected woylies at KWS were infected with *T. vegrandis*. Detailed observations from the captive colony of woylies at NAR suggest that for an established infection, the parasitaemia of *T. copemani* was temporally inconsistent during the longevity of infection. The molecular identification of *T. copemani* in the peripheral blood of the woylie declined over time, with the more virulent strain of *T. copemani* appearing to regress at a faster rate than the less virulent strain. A similar temporal decline was not evident with *T. vegrandis*.

The more virulent *T. copemani* P2 (which corresponds to Clade A Genotype 2 [6]) appeared to decline at a faster rate than *T. copemani* P1 (Figure 4). This more virulent strain of *T. copemani* is genetically and morphologically distinct to *T. copemani* P1 and has been linked with pathological changes and tissue degeneration to the muscles of infected woylies, with reported characteristics being similar to those of Chagas disease in humans [5,6]. The small sample size of this study (P1 (N = 5); P2 (N = 3)) limited our statistical power to detect a significant difference between these two phenotypes, with further research required to investigate the significance of this relationship.

The temporal reduction of *T. copemani* in the peripheral blood may be indicative of an infection transitioning from the acute to chronic phase, after which, the molecular detection of trypanosomes may become inconsistent. During the acute phase of infection, when parasitaemia in the peripheral blood is high, two different morphological forms of *T. copemani* are identifiable in the woylie: broad and slender trypomastigotes [6]. No divisional stages of this parasite have been observed in the peripheral blood of the woylie, despite an extensive morphological investigation [6]. Dividing trypomastigotes, which are essential for maintaining the infection within the woylie, may be confined to the internal organs of the host, similar to *Trypanosoma grosi* within the Mongolian jird (*Meriones unguiculatus*), where dividing forms have been identified within the capillaries of the kidneys long after trypomastigote detection ceased from the peripheral blood [27].

Inconsistent detection of the parasite from the peripheral blood, as was observed in this study, would appear to coincide with transition to the chronic phase, with localisation of these trypanosomes in the capillaries and/or cells associated with the internal organs. The chronic localisation of trypanosomes has previously been demonstrated for the woylie; a molecular analysis of three deceased individuals identified trypanosomes associated with the tissues of the internal organs, while being absent from the peripheral blood [5]. In the current study, both *T. copemani* and *T. vegrandis* were identified from tissue samples, including the heart, bone marrow, kidney, brain and spinal cord; with the latter two organs being identified positive for the first time. While *T. copemani* was not identified from the oesophagus or skeletal muscle in this study, a predilection for these organs has previously been demonstrated from other woylies [5]. The localisation of trypomastigotes within the internal organs of the woylie may be a very important phase for maintaining infection within the host, but as a consequence may be chronically pathogenic, adversely affecting the fitness and coordination of the host.

The association of *T. copemani* with the capillaries and/or cells of internal organs could be responsible for digestive manifestations identified from woylies of ill health. In a newspaper article from ‘The Western Mail’ in 1930, Mr. T. Smith of Kalgoorlie commented that he could assure that disease did get amongst the kangaroo rat [woylie], killing them off in great numbers, with dying individuals having growths in their throats that appeared to interfere with the ability to swallow [28]. These digestive growths identified by Mr T. Smith could have resulted from the same strong inflammation processes identified during histopathological examinations of tissues from the oesophagus and tongues of woylies infected with *T. copemani*[5]. Much like the demonstrated capability of *Trypanosoma lewisi* to influence population change on Christmas Island (*Rattus macleari* and *Rattus nativitatis*), [11-13] the association of *T. copemani* with the recent woylie decline may be another similar case. However, further research is needed to investigate whether the histopathological association of *T. copemani* with the internal organs of the woylie has altered the long term health of the host and influenced the recent decline, or is a result of other, as yet unidentified, stressors. Future investigations will need to correlate the changes of woylie health (over multiple generations and from several different populations) with pathological results, such as haematopathology, histopathology and clinical pathology.

*Trypanosoma copemani* has a broad host range, which also includes the Gilbert’s potoroo (*Potorous gilbertii*), quokka (*Setonix brachyurus*), koala (*Phascolarctos cinereus*), common wombat (*Vombatus ursinus*), common brush-tailed possum (*Trichosurus vulpecular*), tiger quoll (*Dasyurus maculates*) and southern brown bandicoot (*Isoodon obesulus*) [5,29-32]. Its geographic range is also the largest of the native Australian trypanosomes and includes WA, Queensland, New South Wales, Victoria and possibly Tasmania [3,5,6,8,29-34]. If *T. copemani* has had a chronic effect upon the fitness and coordination of the woylie, and has been influential during the recent declines, then it is reasonable to question whether this same parasite has inflicted similar pathological changes and tissue degeneration in its other host species.

In 2008, the initial temporal study that investigated trypanosomes from the UWR woylies reported an overall prevalence of infection of 35% (CI_95_ = 28 - 42%) for samples collected between autumn 2006 and spring 2007 [3]. However, a limitation of the single molecular protocol used in 2008 was that trypanosome co-infections were undetectable. Since then, three separate molecular protocols have been developed, which can independently identify *T. vegrandis, T. copemani* and *T.* sp. H25 [5]. Using these new molecular protocols, the samples collected by Smith *et al.*[3] were re-analysed, and were pooled with UWR samples collected between autumn 2008 to spring 2009 (Botero pers. comm.); the overall prevalence of trypanosome infection from the UWR woylies had increased to 88% [5]. Using the same updated molecular protocols, we have identified a drop in the trypanosome prevalence since 2010, to approximately 33% (CI_95_ = 29 - 37%) for the UWR woylies (where UWR = KP + PP).

This temporal fluctuation of trypanosome infected woylies from the UWR between 2006 and 2012 appears to be of particular importance, as these data sets could provide the necessary link connecting trypanosomes as the chronic disease agent associated with the recent woylie decline. It has recently been identified that woylies within the same region (KP woylies) underwent a period of recovery between 2005 and 2008, before suffering a second decline that began in 2009 [10]. The recovery of the KP woylies between 2005 and 2008 corresponds to the relatively low trypanosome prevalence of UWR woylies as demonstrated by Smith *et al.*[3]; while the timing of the second decline corresponds to the relatively high trypanosome prevalence of the UWR woylies as demonstrated by Botero *et al.*[5]. Following this second decline, the overall trypanosome prevalence from the woylies in the UWR has returned to a relatively low prevalence, as reported here.

**Figure 6:**
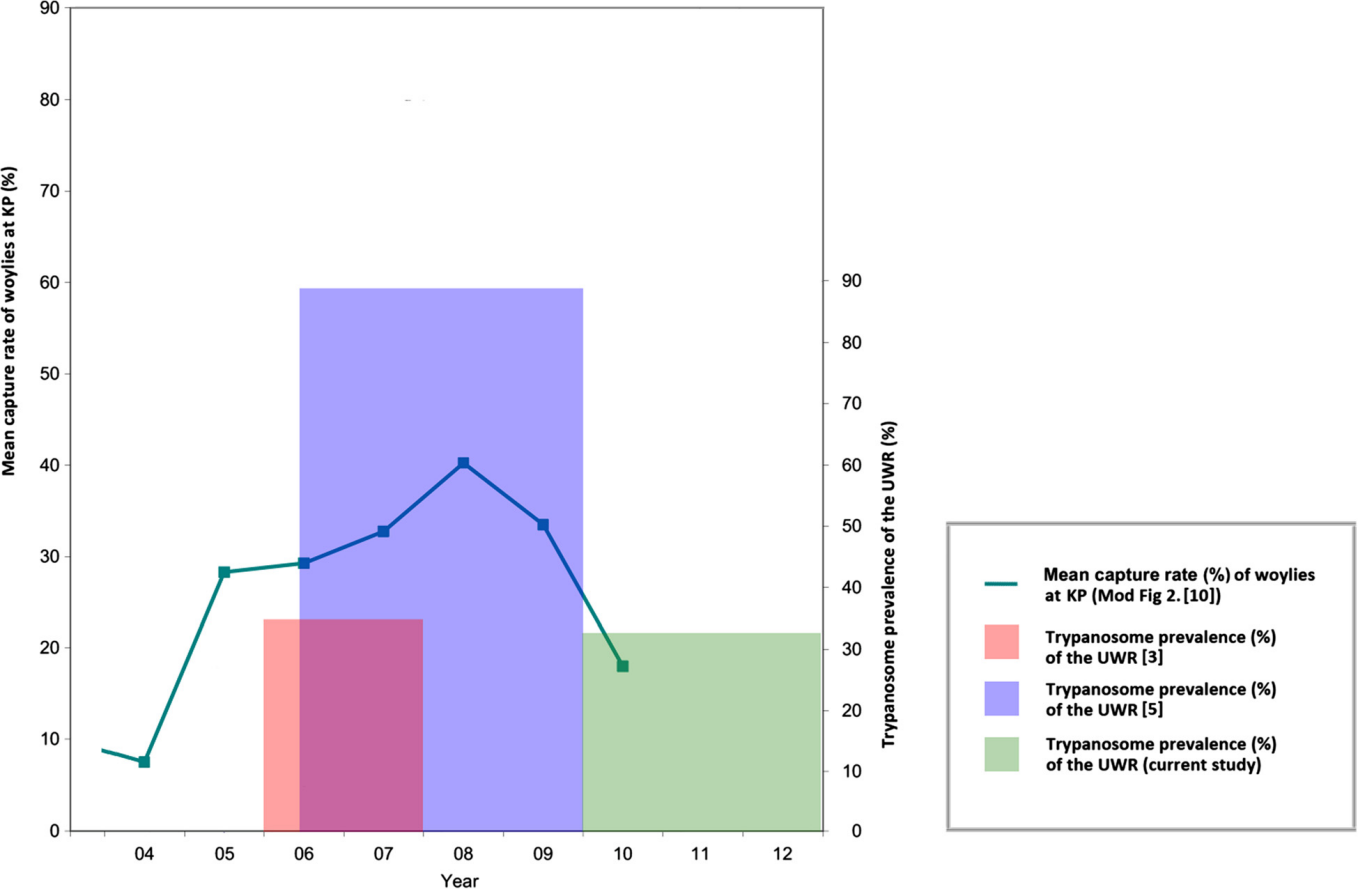
**Changes in the trypanosome prevalence at the UWR and the changing woylie population status at Warrup, (which is part of the KP).** (reproduced with permission from [10]).

From the available data, it appears that trypanosome prevalence in the UWR peaked at a similar time to the second population decline of the KP woylies. When considering the potential of *T. copemani* to localise in the internal organs, causing inflammation and tissue degeneration [5], it may be possible that trypanosomes have affected the long term health, coordination and fitness of these woylies, thus increasing their susceptibility to predation (or to the other, as yet unidentified, stressors). If so, this could provide a temporal link implicating trypanosomes as the disease agent associated with the woylie decline. Figure 6 graphically represents the changing annual trap capture rates of woylies from Warrup (which is part of the KP [10]) and the overall trypanosome prevalence of the UWR (KP + PP) [3,5]. However, it must be noted that due to the spatial variation of the trypanosomes infecting the KP and PP woylies within the UWR, we can no longer assume a uniform distribution of parasites throughout this region. Because of these spatial differences and the incomplete spatial correspondence in this current comparison between woylie abundance and trypanosome prevalence (Figure 6) it is important that the historical samples collected from the UWR between 2006 and 2009 be re-analysed, with samples separated for each of the two neighbouring populations within the region. Through this re-analysis, the role of *T. copemani* as a potential disease agent involved in the recent woylie declines could be clarified.

As evident in this study, the KP woylies had very dissimilar trypanosome prevalences to its neighbouring population (PP) within the UWR. There are four possible reasons for the very low prevalence from the KP. Firstly, the remaining KP woylies are more resistant to trypanosome infections (and the number of woylies in this population should increase); or they are all chronically infected and trypomastigotes are being maintained at molecularly undetectable levels in the peripheral blood of the host (and number of woylies in this population should continue to decline). The very low prevalence of trypanosomes could be a function of woylie density and/or time since the decline (KP woylies declined earlier than PP [10]); or finally, the distribution of the vector has recently changed and these woylies are no longer exposed to infection. Of these explanations, the latter appears unlikely, even though very little is known about the vectors of Australian trypanosomes [2,8,14,35,36]. During this study, the KP and PP woylies that we translocated to the PS enclosure experienced different rates of infection within the same environment, with 11% of uninfected KP woylies later testing positive, compared to 50% of PP woylies. The reason for these contrasting prevalences of trypanosome infections between the neighbouring indigenous populations within the UWR requires further investigation.

Interspecific competition may exist between *T. vegrandis* and *T. copemani,* whereby an established *T. vegrandis* infection may moderate the sequential establishment of *T. copemani*. Co-infections of *T. vegrandis* and *T. copemani* were identified, however, in each case the individual was either initially infected with *T. copemani* and later tested positive to *T. vegrandis* as well*;* or was initially uninfected and then tested positive to both *T. vegrandis* and *T. copemani* simultaneously; or tested positive to both *T. vegrandis* and *T. copemani* during the initial blood sampling (with the sequential acquisition of the two parasites unknown, as it occurred before the initial sample). In this study, all of the woylies that initially tested positive to *T. vegrandis* were never identified later with a co-infection of *T. copemani.* If interspecific competition does exist between *T. vegrandis* and *T. copemani*, then this may be another example where the initial non-virulent infection (*T. vegrandis*) may protect the individual from the sequential infection of a more virulent species (*T. copemani*), as has been demonstrated for other parasite species [37,38].

This interspecific competition could be of particular relevance to the KWS woylies, especially as this fenced sanctuary contains the last high-density stable population of woylies remaining on mainland Australia [17]. The KWS woylies have remained stable, despite an increasing overall prevalence of trypanosomes over the last six years; from 14% reported in 2008, to 46% for samples collected prior to 2010 and up to the current prevalence of 78% [3,5]. The long-term stability of the KWS woylies may be attributed to the high incidence of *T. vegrandis,* which may have kept the chronically-pathogenic *T. copemani* at a very low prevalence. Of those individuals infected with trypanosomes at KWS, 100% were infected with *T. vegrandis* (Table 3), of which only a small proportion were also co-infected with *T. copemani.* No individual woylie at KWS had a single infection with *T. copemani*, which is in stark contrast to the declining UWR. KWS has the lowest prevalence of *T. copemani* from the five populations analysed in the study (Table 3), with further work required to sub-phenotype/sub-genotype this parasite within the KWS population.

## Conclusion

This study highlights the potential negative impact of *T. copemani*, and its possible association with the recent decline of indigenous woylies in WA. In this study, we demonstrated the variable spatial prevalence of *T. copemani* among the five study sites, and the declining molecular detection of *T. copemani* from the peripheral blood of the woylie. The reduction in parasitaemia of *T. copemani* over time may indicate the transitioning of the infection from the acute to chronic phase. We also highlight that the fluctuating trypanosome prevalence of the UWR between 2006 and 2012 could have been influential during the population changes reported for one of the two indigenous populations within this region. Here we add to the growing evidence that trypanosomes could have been influential during the recent declines of indigenous woylies in WA. The associated degenerative pathology from the localisation of *T. copemani* in the capillaries and/or cells of the internal organs may be chronically pathogenic, adversely affecting the long term fitness and coordination, and making the host more vulnerable to predation. We also highlight the necessity to continue monitoring remaining woylie populations, both in the wild and in captivity, and to more thoroughly and rigorously test the nature and strength of the association between trypanosomes and population changes of the woylie and other host species.

## Competing interests

The author(s) declare that they have no competing interests.

## Authors’ contributions

CKT, AFW and RCAT designed the study; CKT, AFW, SSG and RCAT implemented the study; CKT managed the data; SSG and CKT analysed and interpreted the data; CKT wrote the paper. CKT, AFW, SSG and RCAT supervised the different phases of the study. All authors read, revised and approved the final manuscript.
